# Pharmaco-EEG of antipsychotic treatment response: a systematic review

**DOI:** 10.1038/s41537-023-00419-z

**Published:** 2023-12-09

**Authors:** Marco De Pieri, Vincent Rochas, Michel Sabe, Cristoph Michel, Stefan Kaiser

**Affiliations:** 1 Division of Adult Psychiatry, Department of Psychiatry, University Hospitals of Geneva, 2, Chemin du Petit-Bel-Air, CH-1226 Thonex, Switzerland; 2 Functional Brain Mapping Laboratory, Department of Basic Neurosciences, University of Geneva, Geneva, Switzerland

**Keywords:** Biomarkers, Neural circuits

## Abstract

Response to antipsychotic medications (AP) is subjected to a wide and unpredictable variability and efforts were directed to discover predictive biomarkers to personalize treatment. Electroencephalography abnormalities in subjects with schizophrenia are well established, as well as a pattern of EEG changes induced by APs. The aim of this review is to provide a synthesis of the EEG features that are related to AP efficacy, including both pre-treatment signatures and changes induced by APs during treatment. A systematic review of English articles using PubMed, PsychINFO and the Cochrane database of systematic reviews was undertaken until july 2023. Additional studies were added by hand search. Studies having as an endpoint the relationship between AP-related clinical improvement and electroencephalographic features were included. Heterogeneity prevented a quantitative synthesis. Out of 1232 records screened, 22 studies were included in a final qualitative synthesis. Included studies evaluated resting-state and task-related power spectra, functional connectivity, microstates and epileptic abnormalities. At pre-treatment resting-state EEG, the most relevant predictors of a poor response were a change in theta power compared to healthy control, a high alpha power and connectivity, and diminished beta power. Considering EEG during treatment, an increased theta power, a reduced beta-band activity, an increased alpha activity, a decreased coherence in theta, alpha and beta-band were related to a favorable outcome. EEG is promising as a method to create a predictive biomarker for response to APs; further investigations are warranted to harmonize and generalize the contradictory results of reviewed studies.

## Introduction

Antipsychotic medications (APs) are serotonin and/or dopamine receptor ligands exerting a widespread modulatory effect on the cerebral cortex and on the dopaminergic and serotoninergic subcortical projective systems. APs represent the cornerstone in the treatment of patients with schizophrenia (SZ) spectrum disorders and are among the main options for bipolar disorder and treatment-resistant depression^1,2^. Response rates to APs for SZ range from 47% for individuals who have received prior treatments to 66% for antipsychotic-naïve individuals^3^ while 1-year discontinuation rates reach 74% due to poor tolerability and/or efficacy^4^. For this reason, efforts based on several fields of experimental medicine (e.g., genomics, proteomics, neuroimaging) were directed to discover predictive biomarkers to anticipate therapeutic failures, but have so far been unsuccessful^5,6^.

Electroencephalography (EEG) is a neuroimaging technique, with very high temporal resolution and good spatial resolution of the whole brain activity caught in its essential nature of an ensemble of electrical oscillations^7^. In fact, quantitative EEG measures have been associated with many cognitive, motivational, sensorimotor, and emotional processes^8–16^

Typical EEG patterns were defined for many mental disorders; for SZ they include changes in power spectra in multiple frequency bands^17–34^, functional connectivity^18,35–41^, symmetry of the signal^42–48^, microstates (i.e. successive short time periods during which the configuration of the scalp potential field remains semi-stable)^49^ and evoked potentials (i.e. averaged electrical potentials aligned to repeated presentations of a stimulus)^50–52^. These EEG changes were also related to at-risk mental states and early stages of SZ^53^.

A general pattern of AP-induced EEG changes was described with the common features of an increase in delta and theta spectral-power^20,54–60^. Moreover, increased and decreased functional connectivity in AP-medicated patients were discovered in widespread brain areas across all frequency bands^61^. APs can be divided into first-generation APs, whose therapeutic effect is exerted through an antagonism to D2 dopamine receptor, second-generation APs, whose effect is also due the binding to serotonin receptors 5HT2A and 5HT1A and third-generation APs, characterized by a partial agonism to D2 and D3 dopamine receptors^62^. EEG changes specifically related to each category were described. Low-potency first-generation APs provoked a decrease of alpha and an increase of delta, theta and, less consistently, fast-beta activities. On the contrary, high-potency first-generation APs increased alpha and low-beta and decreased fast-beta frequencies^58,63–68^. However, these findings are contradictory, since other studies indicated an overall decrease in delta^63,69,70^ theta and alpha^69^ power and that chronic treatment induce a slowing of delta activity^20^. Concerning specific APs, Clozapine (CLZ) administration increased delta, theta and fast-beta and decreased alpha and slow-beta power in the resting-state^71–76^. An increased amplitude of the P300 component in evoked potentials was reported as well^77,78^. Olanzapine induced a similar array of changes, with an increase in theta and a decrease in alpha and beta power^72^. Concerning gamma frequencies, one report described that an atypical AP normalizes 30-50 Hz low-gamma power^79^, however, another study found no significant relationships in this respect^80^.

Overall, there is solid evidence for EEG alterations in patients with SZ and for AP-induced changes. However, the application of these findings to the clinic remains limited. As outlined above, one of the central challenges in the treatment of SZ and bipolar disorder is the prediction of AP response. Although several studies have addressed this research question, the literature remains limited and has to our knowledge not been systematically reviewed.

**Fig. 1:**
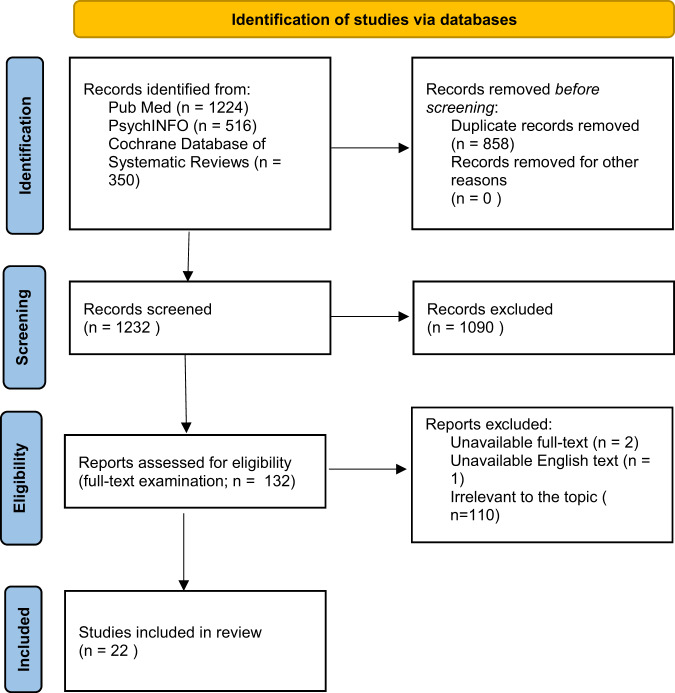
PRISMA flowchart of included studies. PRISMA 2020 flowchart diagram.

The aim of the present paper was to systematically review all the EEG features related to AP efficacy, including both pre-treatment and during-treatment recordings. The final target was to gain an insight over the possibility for EEG to become an instrument useful to personalize AP treatment (Fig. 1).

## Methods

### Study design

The Preferred Reporting Items for Systematic Reviews (PRISMA) statement has been followed to design and conduct the systematic review. We performed a comprehensive literature search on EEG signatures predicting the response to APs or associated to AP treatment outcome. A review protocol was enregistered in PROSPERO (CRD42023450068).

### Article search strategy

A systematic literature search was conducted in three electronic databases: PubMed, PsycINFO and Cochrane Database of Systematic Reviews since inception to 31^st^ July 2023 with no time limit and with English language as the only selected filter.

The following combination of search terms was used:

(“EEG” OR “electroencephalography” OR “Pharmaco-EEG” OR “EEG microstate” OR “dipole source localization” OR sLORETA OR LORETA OR eLORETA OR ERP OR “event-related potential” OR “spectral analysis” OR “frequency domain analysis” OR “spectral band” OR “neural oscillations” OR “spectral power” OR N100 OR N1 OR MMN OR “mismatch negativity” OR P300 OR P3a OR P3b OR “event-related” OR “evoked potential” OR “evoked-response”) AND (“antipsychotic*“ OR “clozapine” OR “risperidone” OR “olanzapine” OR “quetiapine” OR “paliperidone” OR “amisulpride” OR “aripiprazole” OR “brexpiprazole” OR “cariprazine” OR “lurasidone” OR “ziprasidone” OR “haloperidol” OR “zuchlopentixol” OR “chlorpromazine” OR “perphenazine”) AND (“response” OR “efficacy” OR “outcome” OR “effectiveness” OR “efficiency”).

### Selection process and criteria

Firstly, any duplicate from the combination of the three databases was excluded. The remaining articles were included in the systematic review only if they met the following criteria:

#### Inclusion criteria

1. meta-analysis, reviews, clinical trials, case-control and cohort studies;
2. studies carried out in humans;
3. studies published in English;
4. studies conducted on patients affected by a disorder included in the DSM5 chapter “schizophrenia spectrum disorder” and/or “bipolar disorders”;
5. studies reporting on the relationship between EEG and AP efficacy, including both EEG features of pre-treatment recordings predicting response, and EEG features in a during-treatment recording, related to treatment outcome.

#### Exclusion criteria

1. books chapters, comments, editorials, case reports, theses, proceedings, letters, short surveys, notes;
2. studies irrelevant to the topic;

Two researchers (MDP, VR) independently screened for eligibility all the articles by titles and abstracts and then proceeded to read the full text. Any disagreements were resolved by consensus or by the decision of a third reviewer.

### Data extraction

MDP and VR recorded the following variables from each included article: author/s, year of publication, socio-demographic and clinical features, assessment instruments for diagnosis, current and past pharmacological treatments, EEG methods and indexes and EEG data results (Tables 1–3).

Risk of bias was assessed using the Risk of Bias Assessment tool for Non-randomized Studies (RoBINS)^81^, including six domains: selection of participants, confounding variables, intervention measurements, blinding of outcome assessment, incomplete outcome data, selective outcome reporting. We classified studies as having low risk of bias if none of these domains was rated as high risk of bias and three or fewer were rated as unclear risk; moderate if four or more were rated as unclear risk; and all other cases were assumed to have high risk of bias.

The Assessing the methodological quality of systematic reviews (AMSTAR 2 checklist)^82^ was used to assess the quality and completeness of data.

**Table 1:** Sociodemographic and clinical features.

Reference	*N*	Diagnosis	Age	Sex (F)	Education level	Duration of illness (months)	Age of Onset	Rating scale	Baseline severity
*Itil 1981*	13	SZ	49.4	0	Na	276	Na	CGI, BPRS	Na
*Ulrich1988*	34	SZ	32.4 ± 12.1	14	Na	Na	25 ± 9.3	BPRS, SZ-specific sub-score	18.1 ± 7.78
*Czobor 1991*	34	SZ, SA	33.8 ± 7.1	Na	Na	144 ± 72	Na	BPRS	51.1 ± 9.8
*Galderisi 1993*	29	SZ	25.3 ± 4.7	12	8.6 ± 3.5 years	48 ± 36	21.2 ± 3.8	SANS, SAPS, CPRS	Na
*Lacroix 1995*	20	TRS	37.2 ± 7	10	Na	132 ± 60	Na	BPRS	71.15 ± 13.92
*Risby 1995*	16	SZ, SZ	Na	5	Na	Na	Na	BPRS	Na
*Pillay 1996*	86	SZ, SA, BD, DM, others	36.15 ± 10.98	48	Na	Na	Na	CGI, GAF	Na
*Merlo 1998*	13	FEP	23.4 ± 6.2	7	Na	Na	Na	AMDP	Na
*Knott 2000*	17	TRS	35.6 ± 8.9	1	11 ± 3 years	180 ± 84	21 ± 4.5	PANSS	Na
*Kang 2001*	10	TRS	31 ± 6.72	2	Na	Na	Na	BPRS	32 ± 9.1
*Gross 2004*	16	SZ	32.6	9	Na	120 ± nd	Na	PANSSN	30.3 ± 8.1
								PANSSP	19.3 ± 5.8
								PANSSG	53.6 ± 11.4
*Kikuchi 2005*	16	SZ, SZD	27.3 ± 8.2	8	13.8 ± na years	26.6 ± 42.4	Na	BPRS	52.6 ± 14.8
*Wichniak 2006*	64	SZ	26.76 ± 7.84	27	Na	36 ± 60	Na	Clinical judgement	/
*Sumiyoshi 2006*	5	SZ	39.2 ± 10.8	1	13.6 ± 2.2 years	168 ± 84	Na	BPRS	14.6 ± 13.6
								AVLT	19.8 ± 10.5
								GAF	55 ± 6.1
*Kikuchi 2007*	21	SZ, SZD	29.5	10	Na	23.6	Na	BPRS	56.2
*Khodayari-Rostamabad 2010*	37	TRS	39.12 ± 9.3	17	*3.2 ± 1.48	Na	21.2 ± 5	PANSS, QCA	Na
*Ravan 2014*	47	TRS	37.3 ± 9.44	18	Na	Na	Na	PANSS	Na
*Mitra 2015*	15	SZ	28.87 ± 6.81	3	*1 13.3% 2 46.7% 3 6.7% 6 33.3%	55.13	Na	PANSS	83.87 ± 15.9
								PANSSP	27.13 ± 7.46
								PANNSN	21.6 ± 10.3
								PANSSG	35.6 ± 8.26
*Masychev 2020*	62	TRS	37.3 ± 8.9	25	*3 ± 0.78	Na	19.75 ± 2.75	PANSSN	30.72 ± 4.79
								PANSSP	27.93 ± 5.8
								PANSSG	60.45 ± 8.4
*Arikan 2021*	24	BD	36.75 ± 13.09	13	Na	Na	Na	YMRS	6.26 ± 7.58
*Ciprian 2021*	57	TRS	36.95 ± 922	20	Na	Na	Na	PANSS	Na
*Dominicus 2023*	62	FEP	23.2 ± 4.7	29	*1 0% 2 9.7%, 3 61.3%, 4 19.4%	11 ± 14.3	Na	PANSS	74.53 ± 16.97
								PANSSN	27.5 ± 9.01
								PANSSP	18.47 ± 6.99
								PANSSG	18.57 ± 4.38

*N* number of patients, *Na* data not available, / not-applicable, *AMDP* Association of Methodology and Documentation in Psychiatry, PANSS positive and negative syndrome scale; *PANSSG* PANSS general psychopathology scale, *PANSSN* PANSS negative scale, *PANSSP* PANSS positive scale, *BPRS* brief psychiatric rating scale, *YMRS* Young mania rating scale, *AVLT* auditory verbal learning test, CGI clinical global impression; *GAF* global assessment of functioning, *SANS* scale for the assessment of negative symptoms, *SAPS* scale for the assessment of positive symptoms, *CPRS* comprehensive psychopathological rating scale, *QCA* quantitative clinical assessment questionnaire, *TRS* treatment-resistant schizophrenia, *BD* bipolar disorder; *FEP* first episode of psychosis, *SZF* schizophreniform disorder, *SA* schizo-affective disorder, *MD* major depression. Baseline severity of disease is expressed as the score of the corresponding rating scale, *education level expressed in categories: 1 grade 6 or less, 2 grade 7 to 12 without graduating, 3 graduated high-school, 4 admission to college, 5 graduate 2 years college, 6 graduate 4 years college, 7 part graduated/professional school, 8 completed graduated professional school.

**Table 2:** Antipsychotic treatment.

Reference	Medication	Dose	Definition of response	Response rate	Duration of treatment	Co-medications	Previous antipsychotics
*Itil 1981*	Molindole	20	Na	42%	1 week each test	No	Long-acting fluphenazine Long-acting pipothiazine
	Thiothixene	20					
	Fluphenazine	10					
*Ulrich 1988*	Perazine	150	66% score decrease	59%	28 days	Na	Na
*Czobor 1991*	Haloperidol	Na	50% score decrease	Na	Na	Na	Na
*Galderisi 1993*	Clopenthixol	3	50% score decrease	Na	Single dose	No	Na
	Haloperidol	5					
*Lacroix 1995*	Clozapine	25 ± 75	<30% or >35% score change	50%	Na	Benzodiazepines procyclidine	Various APs, 850.4 ± 574.8 mg CPZ equivalent dose
*Risby 1995*	Clozapine	373.4 ± 99	Any rating scale score improvement	/	374 ± 177.4 days	Na	At least 2 AP attempts, minimum dosage 1000 CPZ equivalents/day
*Pillay 1996*	Clozapine	Na	Any rating scale improvement	Na	Na	Na	Na
*Merlo 1998*	Na	Na	30% score decrease	38%	Na	Na	no
*Knott 2000*	Clozapine	381.25 ± 124.33	20% score decrease	84.6%	6 weeks	Na	Various APs
*Kang 2001*	Clozapine	100–350	20% score decrease	40%	4 weeks	Na	Na
*Gross 2004*	Clozapine	50–800	/	/	18 weeks	Na	Various first-generation APs
*Kikuchi 2005*	Haloperidol	1-12	Na	Na	8 weeks	Biperidene Diazepam Pimozide Estazolam Flunitrazepam perospirone	Na
	Risperidone	2					
	Levemepromazine	25-80					
	Nemonapride	9					
	Perphenazine	8					
*Wichniak 2006*	Olanzapine	14.8 ± 6.4	Clinical judgement + continuation of OLZ monotherapy + AP switch not due to side effects	Na	Na	Risperidone Perazine Perphenazine Levomepromazine Flupenthixol Haloperidol Promethazine Sulpiride zuchlopenthixol	Na
	Olanzapine + BZD	15.4 ± 4.3					
	Olanzapine + another AP	15.9 ± 5.8					
*Sumiyoshi 2006*	Olanzapine	3.6	Na	Na	6 months	bromazepam	Haloperidol Risperidone Risperidone+perospirone
*Kikuchi 2007*	Various	175 CPZ equivalents/day	20% score reduction	50%	2–8 weeks	Other FGA or SGA Benzodiazepines anticholinergics antihystaminics	Na
*Khodayari-Rostamabad 2010*	Clozapine	364.2 ± 139.27	50%, 30% or 25% score reduction	50% or 30%	Na	Na	Na
*Ravan 2014*	Clozapine	347	35% score reduction	42%	Na	Na	Na
*Mitra 2015*	Olanzapine, risperidone, haloperidol	At 4 weeks 516.62 ± 208.6 CPZ equivalents; At 8 weeks 496.26 ± 242.35 CPZ equivalents	b/	/	8 weeks	Na	Na
*Masychev 2020*	Clozapine	375.8 ± 121.26	35% score reduction	48.4%	359.2 ± 171.75 days	Na	Na
*Arikan 2021*	Aripiprazole	Na	50% score reduction	58 %	6.26 ± 4.78 weeks	No	Na
*Ciprian 2021*	Clozapine	347	40% score reduction	33.3%	1.4 yrs	Na	Na
*Dominicus 2023*	Aripiprazole	10 ± 5.9	/	/	4-6 weeks	Fluoxetine (20 mg *n* = 1) Zopiclone (7.5 mg *n* = 4) Codeine (2.5 mg *n* = 1)	No
	Amisulpride	285 ± 170.9					

*Na* data not available, / not applicable, *AP* antipsychotic, *BZD* benzodiazepines, *CPZ* chlorpromazine.

^a^a linear relationship between PANSS score and theta power is the measure considered;

^b^correlation between PANSS and frequency-band power is the measure considered.

**Table 3:** EEG methods and statistics.

Author year	Study design	EEG recording	Montage	Paradigm	EEG measures	Statistic test
*Itil 1981*	Random order crossover placebo controlled, following washout	Pre-treatment and after 3 hours	Na	Resting-state and evoked potential (listening to 100 msec 1000 hz tones at 60 dB, every 2 sec)	Absolute power spectra; Time spent in each frequency band	Na
*Ulrich 1988*	Cohort	Pre-treatment, 2 h after test dose, 4 h and 4 weeks of treatment	8 ch	Resting-state (13 min)	Relative alpha power compared to total power spectra	2-way ANOVA, Moore and Wallis trend test
*Czobor 1991*	Double-blind crossover, placebo controlled	Pre-treatment, 3 and 6 weeks of treatment	19 ch, 10-20 system	Resting-state (10-15 min)	Relative power spectra	Multiple regression (with disease severity and prn drugs)
*Galderisi 1993*	Cohort, following washout/drug naïve patients	Pre-treatment and 3, 6, 8 hours after test-dose	21 ch	Resting-state	LAP and LRP power spectra	Wilcoxon, Mann-Whitney U-test
*Lacroix 1995*	Cohort	Pre-treatment and during treatment	19 ch, 10–20 system	Resting-state	Averaged cross power spectra squared root of power amplitude; coherence	Wilcoxon, Logistic regression
*Risby 1995*	Cohort	Pre-treatment and after 6 months of treatment	21 ch, 10–20 system	Resting-state	Epileptic abnormalities	Wilcoxon matched pairs signed rank-test, Mann-Whitney U test
*Pillay 1996*	Cohort	During treatment	Na	Resting-state	Epileptic abnormalities	Mann-whitney U-test, Pearson correlation coefficient
*Merlo 1998*	Case-control on drug naïve pz	Pre-treatment	19 ch	Resting-state	Squared root of power spectra	ANOVAR
*Knott 2000*	Open label case-control, following washout	Pre-treatment and 1.5 h after AP administration	14 ch, 10–20 system	Resting-state	Intrahemispheric and interhemispheric asymmetry	Pearson product moment correlation coefficient
*Kang 2001*	Cohort		8 ch, 10–20 system	Resting-state	Non-linear (D2, PLE, MCP)	Wilcoxon signed-rank test
*Gross 2004*	Following washout	Pre-treatment and after 1, 3, 10 and 18 weeks of treatment	21 ch	Resting-state	Theta absolute power spectra	Wilcoxon signed-rank, Spearman’s correlation
*Kikuchi 2005*	Cohort, following washout or drug naive	Before treatment	18 ch	Resting-state	CFFB and IAF power spectra	ANOVA, Spearman rank-order correlation
*Wichniak 2006*	Cohort	During treatment	Na	Resting-state (20 min), after hyperventilation and photic stimulation	Epileptic abnormalities	Na
*Sumiyoshi 2006*	Pilot case-control	Pre-treatment	19 ch	Auditory oddball task	P300 CSD, laterality, amplitude	Wilcoxon signed-rank test
*Kikuchi 2007*	Case-control	Pre-treatment and after 2-8 weeks of treatment	16 ch, 10–20 system	Resting-state	K-mean clustering algorithm for microsites, GFP, GFS	Pearson’s correlation coefficients
*Khodayari-Rostamabad 2010*	Cohort	Pre-treatment	16 ch, 10–20 system, midline discarded	resting state (3 * 3.30 min)	See candidate features, Table 4	Na
*Ravan 2014*	Cohort	Pre-treatment	20 ch, 10-20 system	Auditory odd-ball task	See candidate features, Table 4	Welch t-test, paired student t-test
*Mitra 2015*	Case control, on drug naïve or drug-free pz	Pre-treatment, after 4 and 8 weeks of treatment	192 ch, 10-5 system	Resting state 3 min	Averaged power spectra	Mann-Whithney U-test, ANOVA, Friedman’s test, Spearman’s correlation, Bonferroni correction
*Masychev 2020*	Cohort	Pre-treatment	20 ch, 10-20 system	Resting state	See candidate features, Table 4	Na
*Arikan, 2021*	Cohort	Pretreatment	19 ch, 10-20 system	Resting-state	Average absolute power spectra	Mann-Whitney U- test
*Ciprian 2021*	Cohort	Pre-treatment	20 ch	Auditory oddball task	See candidate features, Table 4	Na
*Dominicus 2023*	Case-control	Pre-treatment	64 ch	Resting-state	See candidate features Table 4	Random forest regression, Mann-Whitney U-test, Bonferroni correction

*Na* data not available, / not applicable or non-realized, *ns* not significant, *Ch EEG* channel, *Cn-CV* consensus nested cross validation, *STE* symbolic transfer entropy, *mRMR* minimum redundancy maximum relevance feature selection algorithm, *PSD* cross power spectrum density, *LCMV* linearly constrained minimum variance, *ANOVA* analysis of variance, *D2* correlation dimension, *PLE* primary Lyapunov exponent, *MCP* mutual cross-prediction, *IAF* individual alpha frequency, *CSD* current source density, *LORETA* low-resolution electromagnetic tomography, *GFP* global field power, *GFS* global field synchronization. *CFFB* conventional fixed frequency band, *IAF* individual alpha frequency, *LAP* logarithmic absolute power, *LRP* logarithmic relative power.

## Results

### Characteristics of the included studies

The combined outcome of the three databases yielded a total of 1731 records, in addition 15 studies were added by hand search. Of the total studies, 858 were duplicates, leaving 1232 articles. After reading titles and abstracts, 1090 were excluded because they were not relevant to the topic or not respecting inclusion and exclusion criteria. The full-text of 132 articles were examined in details. Three studies were excluded due to full-text unavailability^54,83^ or unavailability of English full-text^84^. After the evaluation phase, a total number of 22 studies were finally identified as eligible for inclusion in the current review.

### Resting state EEG

#### Resting-state power spectra

Power spectra are the power distribution of EEG series in the frequency domain: the local electrical power is estimated at each moment, and it is classically divided in the 5 bands delta (0–4 hz), theta (4–8 hz), alfa (8–12 hz), beta (13–30 hz) and gamma (30–100 hz)^7^.

##### Delta-band

In a pre-treatment EEG, Knott et al.^85^ examined indices of interhemispheric and intrahemispheric EEG asymmetry and their relationship to symptoms changes, using combinations of electrodes. Greater reduction in negative symptoms was associated with greater pretreatment intrahemispheric asymmetry between frontal-anterior-temporal and anterior-midtemporal delta ratios (i.e. the power ratio in delta band between the two ensembles of electrodes).

##### Theta-band

In a pre-treatment EEG, Galderisi et al.^86^ found that average theta2 power was reduced in responders compared to non-responders to AP; quite the opposite, Kikuchi et al.^87^ evidenced that response to AP was positively related to before-treatment theta power (both with the analysis of the average global signal and with the analysis of single electrode, where differences were widespread to almost all sites).

Galderisiet al.^86^ realized their study based on the single dose effect hypothesis (i.e. a single AP administration induces measurable quantitative changes of EEG and patients showing the same changes observed in healthy controls have a favorable clinical outcome): 6 hours after AP administration theta power was increased in responders and unchanged in non-responders.

In EEGs recorded during treatment, Kikuchi et al.^87^ showed that theta-band power was increased in responders, irrespective of the scalp position.

In a pre-treatment EEG Knott et al.^85^ found that a higher global intrahemispheric theta asymmetry was positively associated with overall symptoms reduction, and that a greater interhemispheric central-anterior-temporal theta ratio was associated with greater treatment-related reduction in positive symptoms.

Gross et al.^88^ focused on the fronto-central areas theta-band oscillations. After 3 weeks of treatment they detected a significant negative relationship between theta-power change as measured by midline electrodes and PANSS subscales for positive and negative symptoms – so positively correlated to the response to APs. After 10 weeks of treatment, the relationship was widespread to all of the electrodes, but significant only for PANSS-positive symptoms and PANSS general subscales. After 18 weeks this finding persisted for PANSS general and negative symptoms subscales, but not for the positive symptoms’ subscale.

##### Alpha-band

In a pretreatment EEG, Itil et al.^66^ found that nonresponders had an increased power in the alpha range, considering the signal from all over the brain; the study replicated the findings from the same group that was excluded from the review due to full-text unavailability.

Kikuchi et al.^87^ discovered that pre-treatment alpha2 power was positively related to response to APs on the average and at the single-electrode level in P4, T5, T6 and O2.

Czobor et al.^89^ discovered that higher alpha activity on a pre-treatment recording is associated with a poorer outcome at 3 and 6 weeks of treatment; however, the explained variance was low (13% after 3 weeks of treatment and 18% after 6 weeks). In term of localization, this relationship was significant for the anterior and the temporal areas at both 3 and 6 weeks of treatment, and for the posterior areas at 3 weeks only.

Merlo et al.^90^ evaluated the relationship of pre-treatment EEG features with speed of response to APs. Alpha2 band power were higher in early compared to late responders, widespread to almost all the electrode locations.

In a pretreatment recording, Galderisi et al.^86^ found differences concerning alpha band with responders having lower alpha1 and higher alpha2 power compared to non-responders. Six hours after AP administration, alpha1 was increased in responder and decreased in non-responders and alpha2 was unchanged in responders and increased in non-responders. Individual data showed a large overlap between responders and non-responders, except for alpha1 change after 6 hours AP administration, for which an opposite pattern was clearly observed between the two groups. For this reason, this frequency-band only was used for the sensitivity-specificity analysis, and its changes at the electrode C3 resulted as the best discriminant feature of responder and non-responder, including both baseline recording and the one realized after 6 h, with an accuracy of 89.3%.

Ulrich et al.^91^ focused on the individuation of alpha and non-alpha epochs (i.e. relative power spectrum density of alpha-band > or < 50%) in acutely ill, drug-free patients. Both in pretreatment and during treatment recordings, responders had more non-alpha epochs compared to non-responders. A further analysis focused on the dynamic of EEG vigilance (i.e. including time as a variable and thus observing the evolution of the signal along the recording) found a significantly increased density of non-alpha epochs in responders compared to non-responders, both in pre-treatment and during treatment EEG. Differences were mainly localized on bilateral anterior-occipital and anterior-frontal derivations. No other difference in power spectra emerged between responders and non-responders in this investigation.

##### Beta-band

Results concerning beta-band were only found regarding pre-treatment EEG. Two studies^66,86^ found that non-responders to APs had a diminished power in the beta range compared to responders. Moreover, beta band power in almost all regions was higher in patients having and early response to AP, compared to late responders^90^. Knott and colleagues found that a higher interhemispheric central-anterior-temporal asymmetry in the beta band is associated with a better improvement of negative symptoms in the course of the disorder^85^.

##### Gamma-band

Mitra et al.^80^ hypothesized that pre-treatment gamma oscillations would have been higher in SZ compared to HCs, and that there would have been a reduction in their activity over the course of AP treatment. Even if the primary outcome of the study was not to find EEG signatures of AP response, the analysis included correlations between AP efficacy and gamma frequencies, but no significant results emerged in this respect.

In a pre-treatment EEG, Arikan et al.^92^ discovered a lower high-gamma power in responders at multiple frontal, central, parietal, temporal and occipital regions (electrodes FP1, F3, F4, C3, P4, Pz, O1, F7, F8, T4 and T5). In contrast, Itil et al.^66^ found that nonresponders have a diminished gamma-band power in EEG recorded before starting an AP therapy.

##### Other results

Itil et al.^66^ found that non-responders had a higher EEG signal average amplitude and amplitude deviation compared to responders on a pre-treatment recording, considering all the frequencies together.

Lacroix et al.^93^ studied EEG recorded during treatment to assess changes concerning amplitude and coherence of the signal in the main frequency bands. Response to AP was not the primary outcome, but its relationship with resting-state amplitudes was studied finding no statistically meaningful associations. A significant result was found instead for connectivity measures, as described in the paragraph below.

### Resting-state connectivity

EEG functional connectivity is the temporal coincidence of spatially distant neural activities that likely reflects the dynamic interregional communications in the brain, and it is calculated for all the power frequency bands described above^7^.

Comparing pre- and post-treatment EEG, Lacroix et al.^93^ realized a connectivity analysis focused on EEG amplitude and coherence changes induced by CLZ. Changes in coherence in theta were negatively correlated with clinical improvement for couples of electrodes involving right anterior-medial temporal region against the frontal electrodes, and the left-parietal electrodes. A relationship between response and changes in coherence also in the alpha band, concerning left temporal electrodes paired with frontal electrodes. Moreover, in central electrodes, the amplitude of beta1-band was increased in less-responsive compared to more responsive patients, and the former showed a decrease in coherence in this band involving almost all electrodes.

By analyzing during-treatment EEG recordings, Kang et al.^94^ evaluated CLZ-induced EEG changes in functional connectivity, using non-linear methods. A result of their analysis is the subdivision of patients in two categories, one with frontal driving - occipital response (FDOR; i.e. a condition in which frontal regions are the inductors of the activity of the other regions) or one without this pattern. The study also reported that the clozapine induced the non-FDOR group to have a FDOR pattern after treatment and that this group showed a better clinical response, without a statistical measure in support.

### Microstates

Microstates are global patterns of scalp potential topographies recorded using multichannel EEG arrays that dynamically vary over time in an organized manner. Broad-band spontaneous EEG activity at rest can be described by a limited number of scalp potential topographies, that remain stable for 60-120 milliseconds before transitioning to a different topography that remains stable again^49^.

One study reported microstates measurement linked to the response to AP, in a pre-treatment EEG. Kikuchi et al.^95^ found that responders had an overall shorter duration of microstates than controls or non-responders and a shorter duration of microstates A and D; occurrence of microstates B and C and of microstates in general was increased in responders compared to non-responders. Responders also had a lower total duration of microstate D compared to controls. Considering the percent change in microstate parameters induced by treatment (evaluated with a control EEG), responders had an increased duration of microstate A and D and overall microstate duration, a decreased duration of microstate C and reduced overall microstate occurrence compared to non-responders. The study moreover discovered a negative correlation between clinical improvement and duration of microstate D and total microstates duration, and a positive correlation with the occurrence of microstate A, microstate C and overall microstate occurrence. The analysis of global field synchronization (estimate of functional synchronization in the frequency domain) yielded no significant results.

### Evoked potentials

An evoked potential or evoked response is an electrical potential recorded from the nervous system of a human or animal following the presentation of a stimulus. Resting-state analysis only reflects background brain processes, while evoked potentials explore the functioning related to task and active engagement in the environment^7^.

Sumiyoshi et al.^96^ aimed to assess changes in the configuration of the P300 wave generators in an auditory odd-ball task and following a treatment with olanzapine. The results showed an increase of a calculated laterality index of temporal lobe activation following 6 months of treatment; restoring the pattern observed in the control group. However, a significant relationship between this lateralization index and the response to treatment did not emerge, probably due to the small sample size.

### Machine learning approach

Five studies used a machine learning approach to discover predictors of response to CLZ in treatment-resistant schizophrenia, using a pre-treatment EEG (Table 4).

**Table 4:** Machine learning methods and results.

First author year	Training set	Extraction process: candidate features	Features selection	Specification of classifier	Performance Evaluation	Top features
*Khodayari-Rostamabad 2010*	EEG epochs and treatment outcome for each subject	• Coherence between all electrode’s pairs at all frequencies • correlation and cross-correlation coefficients • mutual informations (Cover and Thomas 2006) • absolute and relative power levels • left-to-right hemisphere power ratio • anterior-posterior power gradient across frequencies and between electrodes	Regularized features selection method followed by normalization (Peng 2006)	Kernel partial least square regression procedure (Rospital & Kramer, 2006) Non-linear kernel principal component analysis (Muller 2001)	L1O cross validation procedure	• *Mutual information*: T3/P3; T3/O1; C3/P3 • *Correlation* F8/T4 • *Coherence*: 6hz T3/O1; 6hz T3/P3; 6hz C3/O1; 7 hz F3/P3; 8hz T6/P3; 9hz T3/O1; 10hz T3/T5; 10hz T3/P3; 11hz C3/P3; 11hz T3/P3; 12hz T3/T5; 13hz F7/F3 • *Left-to-right PSD-ratio*: 10 hz T5/T6; 11hz T5/T6; 12hz T5/T6; 16hz T5/T6
*Ravan 2014*	EEG responses for patients and HC and corresponding labels	• auto-PSD values of 15 extracted source waveforms • c-PSD values between all pairs of source waveforms, at various frequencies (with transformation in a decibel scale and normalization)	Regularized features selection (Peng) method followed by normalization	Fuzzy c-mean method (Dunn 1973 & Bezdek 1981)	L1o cross validation procedure (Theodoridis, 2008)	CPSD: (FpM, TAR) at 19hz; (FpM, PR) at 19hz; (FpM, FM) at 16hz; (CM, PR) at 14hz; (OpM, PR) at 2hz
*Masychev 2020*	EEG epochs and treatment outcome for each subject	STE connectivity values (all channels, all frequency bands)	Cn-CV; relief features method; KPCA	SVM, LNA, KNN, RF	Cn-CV algorithm	*Effective connectivity*: δ T3 to P4; θ F3 to T6; θ T6 to F3; α FP2 to Fz; δ T4 to O1; γ F1 to F2; γ F3 to F2; γ C3 to Cz
*Ciprian 2021*	EEG responses to the oddball and corresponding labels, for patients and healthy controls	STE connectivity values (all channels, all frequency bands)	mRMR, CN-CV validation procedure; KPCA	SVM, LNA, KNN, RF	Cn-CV algorithm	*Effective connectivity*: θ right-IFG to left SPL; γ left LC to left LTL; θ right OFC to right ACC; θ left SFC to right PCC; θ left PCC to left AFC; γ left ACC to left IFG; β left FG to right SMA; θ left PCC to left AFC
*Dominicus 2023*	EEG epochs and treatment outcome for each subject	• Relative power spectra • mean phase lag index (PLI) • mean amplitude envelope correlation corrected (AEC-c) • Minimum spanning tree (MST)	Random noise feature	RF	/	δ, Relative power; θ _MST – degree; θ, Relative power; θ _MST – Leaf fraction; α, Relative power; θ _MST – Diameter; β, Relative power; θ _MST – BCmax; δ, PLI; θ _MST – Ecc; θ, PLI; θ _MST – Tree hierarchy; α, PLI; α _MST – degree; β, PLI; α _MST – Leaf fraction; δ, AEC-c; α _MST – Diameter; θ, AEC-C; α _MST – BCmax; α, AEC-C; α _MST – Ecc; β, AEC-C; α _MST – Tree hierarchy; δ _MST_MST – degree; β _MST – degree; δ _MST_Leaf fraction; β _MST – Leaf fraction; δ _MST_Diameter; β _MST – Diameter; δ _MST – BCmax; β _MST – BCmax; δ _MST – Ecc; β _MST – Ecc; δ_MST _Tree hierarchy; β _MST – Tree hierarchy

*Cn-CV* consensus nested cross validation, *L1O* leave one out, *SVM* support vector machine, *LNA* linear discrimination analysis, *KNN* K nearest neighbors, *RF* random forest, *KPCA* Kernelized principle component analysis with a polynomial kernel, *STE* symbolic transfer entropy, *mRMR* minimum redundancy maximum relevance feature selection algorithm, *CPSD* cross power spectrum density, *MST* minimum spanning tree, *PLI* phase lag index, *AEC* amplitude envelope correlation, *BC* betweenness centrality, *IFG* inferior frontal gyrus, *SPL* superior parietal lobule, *LTL* lower temporal lobule, *OFC* orbitofrontal cortex, *SFC* superior frontal cortex, *PCC* posterior cingulate cortex, *AFC* anterior frontal cortex, *SMA* supplementary motor area.

Khodayari-Rostamabad et al.^97^ evaluated resting-state EEG in two groups, used as discovery and replication cohorts, respectively. After the feature extraction and reduction processes, 8 features were applied for performance evaluation. A first step was realized on the discovery cohort, a second step used the discovery cohort as a training dataset, and tested the prediction performance in the replication cohort. A set of features was able to discriminate the responders form non-responders, including measures of mutual information (i.e. assessment of the amount of information about one signal contained in another signal), correlation (i.e. the quantification of the degree of association between apparently different signals), coherence (i.e. the normalized cross-power spectrum per frequency of two signals recorded simultaneously at different sites of the scalp) and left-to-right power spectra ratios involving both hemispheres on central, temporal and occipital areas, in theta, alpha and low-beta frequency-bands. Accuracy in classification of responders and non-responders was of 87.3% in the first step and of 85.7% in the second step.

Masychev et al.^98^, focused on EEG resting state functional connectivity between frequency bands, through measurements of symbolic transfer entropy. Using four different methods for classifier specification, patients were divided in most-responders and least-responders to treatment, and the ML process discovered 8 features effectively discriminating the two categories, consisting of measures of connectivity between frontal, prefrontal, central and temporal electrodes, at multiple frequency bands. The subsequent statistical-power analysis revealed high values of sensitivity and specificity with all the classifier methods. Accuracy in predicting responder status was of 89.9% ± 4.39 with support vectoring machine, of 87.98% ± 4.66 with linear discrimination analysis, of 87.98% ± 4.66 with K nearest neighbour and of 83.97% ± 6.32 with random forest.

The same research group realized another study^99^, with the same design but the EEG-paradigm, consisting in an auditory oddball task, and importantly the features were extracted from source reconstruction prior to ML. The discriminating features that were identified by mean of the ML process were then 8 measures of functional connectivity in the theta, gamma, and beta frequency ranges across multiple brain regions. In this study accuracy was of 95.83% with support vectoring machine, of 93.37% with random forest and of 91.48% with linear discrimination analysis.

Another ML study was realized by Dominicus et al.^100^, who tested a random forest regression model aimed to determine the response to an APs monotherapy after 4-6 weeks, in FEP patients. Sixty EEG features were included in the model, comprising measures of power spectra and functional connectivity in all frequency bands and widespread to multiple bilateral brain regions. The 5 more important features to predict response were the tree hierarchy in alpha band according to amplitude envelope correlation corrected (i.e. the absolute value of the Hilbert transform of a given cortical oscillation), phase lag index (i.e. a measure of the asymmetry of the distribution of phase differences between two signals) in beta band, betweenness centrality (i.e. the fraction of the second shortest path that pass through a node) in delta band, amplitude envelope corrected in theta-band and, tree hierarchy in beta-band according to phase lag index. A significant predictive power emerged only for PANSS positive subscale (*P* < 0.004), but with a low effect size (R^2^ = 0.23). No significant results emerged for PANSS total score and other PANSS subscales.

Ravan et al.^101^ used not only a pretreatment EEG, but also an after treatment recording, in both cases obtained during an odd-ball task. Comparing the two recordings, a decreased value of five measures of functional connectivity between frontal, parietal, temporal, central and occipital areas in beta and gamma bands was related to a better response to clozapine. Accuracy in predicting response to APs was 79.6%.

### EEG epileptiform abnormalities and response to AP

Epileptiform abnormalities are abnormal synchronous electrical discharges generated by a group of neurons in the region of an epileptic focus^102^.

Two studies discovered that pre-treatment epileptiform abnormalities predicted a favorable response to APs. In one case the relationship was established only in patients with a psychotic disorder having a comorbid major depressive episode and in females^103^, in the other for the whole cohort; in this second study a relationship with response was defined also for an EEG repeated after 6 months^104^. A third study on the topic did not find a relationship between the presence of slow EEG activity (i.e., theta activity for more of 10% of a 1 second EEG epoch or delta occurrence) and/or EEG abnormalities, and treatment outcome and/or sleepiness^105^.

## Discussion

Our systematic review illustrates how multiple EEG features are linked to response to APs. Given the heterogeneity of experimental paradigms and considered variables in the eligible studies, we did not carry out a meta-analysis. The EEG features we reviewed can be divided in two types: pre-treatment predictors of response to AP, and EEG changes occurring under treatment and mirroring APs efficacy. These findings support the notion that local and long-range brain network dysfunction underlying psychosis are mirrored by EEG recording, and that the effect of APs on disrupted neurocircuits produces measurable changes in EEG as well.

AP-induced EEG changes are known from previous studies not focused on response to APs or on the relationship with other clinical variables^20,28,54,56,58–60,63–70^; only some of them proved to be indeed related to response to treatment in the studies we systematically reviewed. The other changes could possibly be only an unspecific influence on brain rhythms or mirror CNS side effects of APs.

### Prediction of response to AP with EEG features

In resting-state EEG, most of the findings concerned theta and alpha bands. At baseline theta power, either low^86^ or high^87^ was related to a favorable treatment outcome. A high alpha power predicted a poorer response in most of the studies^66,86,87,89–91^; moreover, alpha functional connectivity in frontal and temporal regions was inversely related to response^93^. A diminished beta-power on a pre-treatment RS-EEG was related to a worse treatment outcome^66,85,86,90,93^, such as a reduced functional connectivity after treatment^93^.

These findings could be read in light of psychosis pathophysiology and APs mechanism of action. Dopaminergic neurons fire either in a tonic way at 2-8 hz firing or in a phasic way, at 20 hz^106,107^, and the mesolimbic dopaminergic dysfunction is a mechanism at the origin of SZ^108^. Findings of a disrupted alpha, beta and theta in SZ could be a manifestation of the abnormalities of dopaminergic neuron firing; being the leverage for action of APs based on a modulation of dopaminergic receptor, it makes sense that these abnormalities can be changed by AP and that it corresponds to their future efficacy. Considering the different patterns of receptor bindings^1^, it could also be expected that the EEG signatures predicting response differ for different specific APs, but the studies under review don’t offer conclusions in this respect.

In gamma band, responders had a larger pre-treatment resting-state power in frontal, central, parietal, temporal and occipital areas^80,92^, while no differences emerged concerning functional connectivity. Gamma frequencies are considered the direct expression of the activity of the parvalbumin-containing GABAergic interneurons^14,109,110^, whose dysfunction is the other plausible pathophysiological process of SZ, based on the glutamatergic hypothesis^2^. Findings show disrupted power-spectra activities in SZ, and an action of APs on these rhythms in relation to the response, and this is in line with this theoretical background.

A smaller interhemispheric asymmetry at multiple frequency bands^85^ predicted a worse response to treatment.

In SZ a reduced asymmetry was defined both at structural^111^ and at the functional^112^ level, and linked to a reduced brain lateralization, especially in language areas. This reduced brain specialization has been proposed as one of the core pathogenetic processes in SZ^113^. Thus, we could speculate that a higher pre-treatment EEG asymmetry corresponds to a lesser brain impairment, facilitating a better response to therapeutics.

Machine learning studies used measures of functional connectivity involving all the frequency bands and across different brain regions as features in their predictive model, both at the resting state or during the execution of auditory tasks^97–101^. The relevance of functional connectivity measurements as an indicator of treatment response is in line with the conceptualization of SZ as a functional disconnection syndrome, whose core mechanism is not localized to a specific brain area or a function but involve large-scale neurocircuitries^37,38,114^.

On one side, the involvement of all frequency bands widespread to most brain regions is in line with a conceptualization of SZ as whole brain disease, linked to large-scale network disruptions^115^. On the other side, the link between frequency bands and the pathophysiology of psychosis is loose, except for gamma frequencies; the absence of a clear functional hypothesis for the other frequency bands concerning SZ contributes to the uncertainty surrounding the findings.

While most of the studies under review evaluated the relationship between general response to AP and EEG features, some^80,88,98,100^ explored the relationship with specific psychopathology domains, corresponding to the PANSS subscales for positive, and negative symptoms and general psychopathology. The fact that certain EEG features only relate to a peculiar category of symptoms indicates once more that SZ is a multidimensional disorder^116,117^. Each dimension possibly has a specific biological substrate in terms of type and location of neurophysiological activity.

Kikuchi et al.^95^ focused their investigation on microstates and observed that several characteristics of all 4 microstates (i.e. occurrence, duration and topography) were related to response to APs, considering both pre-treatment and during-treatment recordings. Of note, a meta-analysis^118^ summarized results on SZ and microstates, discovering that the key feature in SZ were a more frequent occurrence of microstate C and a shorter duration of microstates D compared to healthy control; the study of Kikuchi indicates that this C-D imbalance can be normalized by AP treatment

### Changes in EEG features during treatment

An increased theta power^86,88^, a reduced beta band activity, a decreased coherence in theta, alpha and beta band^88^ were related to a favorable outcome.

Different frequency bands are altered in SZ, as detailed in the introduction; also, they are implicated in brain functions impaired in psychotic disorders. Delta frequency evoked responses play a role in motivation, emotions, and overall cognitive functioning^8,9^. Theta band orchestrates cognitive processes that are compromised in psychotic disorder, such as working memory, detection of new sensory stimuli and attentional control^10^. Neuronal oscillations in the alpha band play a pivotal role in cognition, consciousness, wakefulness, sensorimotor and emotional processing^11^. Beta oscillations were related to sensorimotor behavior, perceptual integration, working memory and top-down regulation of attention^12,13,119^. Gamma frequencies have been associated with sensation, perception, attention, cognitive processing, consciousness, memory and and more widely to synaptic plasticity^14,15,115,120^.

AP-inducing changes in these frequency bands possibly have their therapeutic effect mediated by a re-tuning of brain circuits underlying the sensorial, cognitive, and affective functions above-mentioned.

Only a pilot study evaluated an evoked potential as a marker of response, and suggested that olanzapine was able to restore the normal features of P300, which has a lower amplitude and a prolonged latency in SZ^121^.

### Quality assessment

The AMSTAR checklist^82^ was used to improve the quality of this review. Included studies where described in details (in the text or in the tables) concerning population, intervention, comparators (i.e. control group, different diagnostic groups, same patient on different time points), outcomes (i.e. response to treatment) and research design. Methods were established prior to the conduct of the review, and all the types of studies suitable to investigate the variable of interest (i.e. EEG features related to response to AP) were included. Study selection and data extraction were performed in duplicate. The exclusion of initially retrieved studies was explained. Funding and authoring are clearly reported.

Risk of bias was assessed with RoBINS-I^81^, a tool that is normally used for interventional trial, but also useful to evaluate non-randomized observational trials as the studies under review. Concerning possible pre-intervention bias, we observed a wide variability in factors predicting response to treatment, including age, duration of illness, severity of disease, education level. In some studies, these parameters were not collected or they were not included as confounders in the statistical analysis. Selection of patients was globally correct, including a single diagnosis in each study. The intervention (i.e. the AP administration) was properly classified and reported. Controls subjects, when implicated, were well matched with patients for age, sex and education level. Significant attrition, detection and outcome reporting bias were not detected. Overall, a low to moderate risk of bias was assessed.

### Limitations

Our systematic review was limited by the small number of studies available and by their methodological heterogeneity, which does not allow to draw univocal and generalizable conclusions.

Different rating scales were used to define response to treatment; even if the concordance between them is high^122^, a major limitation is represented by the use of different cut-offs for response, and of different time span for its determination.

EEG measures were heterogeneous between studies, in term of paradigm and analysis methods. Intra- and inter-subject variability of EEG recording was another major concern: EEG studies are generally characterized by a high variability within and even more between subjects, which might not always be indicative of any pathological status, making it difficult to generalize reports without applied normalization. Disparate EEG features were the focus in different studies; as discussed above, each frequency band correspond to distinct sensory, cognitive and emotional processes, both concerning power spectra and functional connectivity. However, the former mostly reflects local brain areas functioning, and the latter the long-range synchronization of distant regions. P300 correlate with memory, attention, auditory discrimination, processing of sequential information, and decision making^123^. Microstates reflects the fundamental functioning of consciousness^49^, of the visual system^124^, of the salience network^125^, of the default mode network^124^ and of the dorsal attention network^49^. Actually, it is possible to conclude that distinct EEG features reflect different aspect of brain functioning, each one having a peculiar relationship to psychopathology domains and being differently influenced by APs. As a consequence, the heterogeneity of finding precludes drawing generalizable conclusions.

Moreover, most of the data concern resting state activity, while only a minority is from task-related EEG, a difference that further prevent a generalization. Finally, even when the same EEG features were evaluated in different studies, different EEG analysis techniques account for heterogeneity in results.

Intersubjective variability in socio-demographic and clinical features such age, sex, educational level, duration and severity of disease was also considerable, and these parameters have been demonstrated to have a significant influence on EEG in patients with SZ^18^. Moreover, relevant prognostic factor/confounders for response to treatment such as education level, duration of illness, duration of untreated psychosis^126,127^ weren’t considered in the statistical analysis, exposing results to a risk of bias.

Overall, most of the studies did not report a significant amount of data about sociodemographic and clinical characteristics of patients.

Some of the features used for the connectivity analysis, such as coherence, correlation and mutual information are highly subject to volume conduction effect (i.e. at the level of each EEG channel, the effect of a mixture of active brain and nonbrain electrical sources whose activities are conducted to the scalp). This phenomenon could produce results wrongly indicating a functional connectivity or hemispheric asymmetries^128^.

Concerning the medication administered, most studies were conducted on clozapine, while the others on many different FGA, SGA and TGA. On one side this allowed to reproduce findings on CLZ, but on the other side deprives of information concerning other APs. Each AP has a peculiar binding profile, depending on intrinsic activity and constant of dissociation from serotonin and dopamine receptors; accordingly, the way they influence glutamatergic pyramidal neurons and dopaminergic neurons is substance specific, and it can be reflected by different EEG patterns. Moreover, the accessory effect of APs on histaminergic and cholinergic systems further differentiate AP and their influence on EEG activity^2,129^. In fact, the generic effect of APs (i.e. not related to response) on EEG is different when comparing different molecules^20,54–80^. This could explain why EEG markers of response are not overlapping from one AP to another.

Many important information about the treatment were often missing, such as dose, duration of treatment, co-medications, previous medications, and when present displayed a large variability among studies and subjects under study.

Some of the studies were specifically focused on treatment-resistant SZ, which represents a specific category of SZ patients, differing from the nontreatment-resistant one^130^.

The statistical analysis in most of the studies was minimal, excluding a sensitivity-specificity analysis and the effect-size determination, precluding a formal evaluation of EEG feature as predictive biomarkers.

Only a part of the studies had a case-control design, the other being cohort studies.

## Conclusions

EEG still is a promising method for brain study and to create a predictive biomarker for response to APs, since it is non-invasive and allows a direct in-vivo observation of brain oscillations. Further investigations are warranted to address the limits of the studies under review; novel studies should have a larger sample size, display a better clinical and demographic characterization of the sample, extensively evaluate all of the APs, use HD-EEG and EEG paradigms including auditory, cognitive and emotional tasks. While the studies reviewed here were mostly of exploratory nature, we believe that the most promising approach for novel studies would be to select frequencies, regions of interest and connectivity measures mirroring a sound theoretical hypothesis on the pathogenesis of SZ and the mechanism of action of APs. If large data sets are available, an alternative to this hypothesis-driven approach could be data-driven approaches based on machine learning.

### Supplementary information

Supplementary table 1
