# Supplementary material for: Pharmaco-EEG of antipsychotic treatment response: a systematic review
Source: Schizophrenia (Heidelb). 2023 Dec 9;9(1):85. doi: 10.1038/s41537-023-00419-z (PMC10710499; doi:10.1038/s41537-023-00419-z)
Supplement: Supplementary file 1 — Supplementary table 1 [file 41537_2023_419_MOESM1_ESM.docx]

**Supplementary table 1**: *other* *results, not concerning response to antipsychotics.*

| Reference | Findings |
| --- | --- |
| *Itil*  *1981* | - only one patient showed a typical neuroleptic response, one showed an anxiolytic response to molindone, one an antidepressant response to fluphenazine, 9 patients a typical psychostimulant response. |
| *Ulrich*  *1988* | / |
| *Czobor*  *1991* | / |
| *Galderisi*  *1993* | / |
| *Lacroix*  *1995* | - Compared to the general population norm (no control group was included) both HR and LR had a significant increase in absolute amplitude in theta and alpha band generalized to all electrodes, and localized centrally for beta-band. - Decrease of amplitude at frontal, central and anterior-medial temporal electrodes in beta2 and beta3 for both HRs and LRs. - HR patients showed a decreased coherence involving right frontal, central and temporal, left central, parietal and midline frontal electrodes in theta band - Reduced coherence widespread to almost all electrodes for HR in alpha-band: prefrontal electrodes (F3, F4), right central (C4), temporal (T4), left central (C3), temporal (T3), parietal (P3) and midline (Fz, Cz, Pz). - In HR patients increased coherence in all bands between fronto-frontal paired electrodes - In HR patients increased coherence in beta-band between anterior-medial temporal (T3, T4) electrodes - Massive decrease in coherence in LR especially in alpha and beta2 bands involving almost all electrodes |
| *Risby*  *1995* | 1. No relationship between CLZ dose and probability to have epileptic abnormalities. 2. No differences in the number of days on clozapine between the subjects who did not develop EEG abnormalities and those who did. |
| *Pillay*  *1996* | / |
| *Merlo*  *1998* | - Power spectra in alpha2 band and beta2 band was higher in patients vs controls. |
| *Knott*  *2000* | - Pretreatment EEG asymmetrie from population norm, involving all four frequency bands:  1. For delta and theta interhemispheric asymmetries were evident at frontal/central and homologous site pairs respectively; 2. For alpha and beta, asymmetry deviations were evidenced at occipital and central/occipital electrodes, respectively;  - All site pairs but one (F4-T6 delta site pair) exhibited significant group average intrahemispheric positive Z-score values; - Increased theta intrahemispheric ratio at frontal-occipital and anterior-temporal-occipital regions were positively associated with greater positive symptoms rating. |
| *Kang*  *2001* | - After the CLZ treatment, the non-FDOR pattern was converted to FDOR in all but one subject. - In all of the subjects with a pre-treatment FDOR pattern, the pattern did not change after 4 weeks of CLZ treatment.   Taken together, all of the subjects tended to show a FDOR pattern after 4 weeks of CLZ treatment. |
| *Gross*  *2004* | - A trend of positive correlation between changes in the PANSS subscales and serum CLZ levels was observed after 1 and 3 weeks and a trend of inverse correlation was seen after 18 weeks of CLZ treatment. - Non-responders showed a midline electric potential significantly inferior to responder after 18 weeks of treatment. |
| *Kikuchi*  *2005* | - Theta power before treatment was smaller than after treatment, while there were no differences in the other frequency bands; - There was no significant correlation between risperidone equivalent dose and percent variation from baseline BPRS score or that of EEG power. |
| *Sumiyoshi*  *2006* | - Temporal regions were activated with a left dominant laterality concerning current source density in all control subjects, while this activation pattern was not evident in patients at baseline; - After 6 months of treatment with olanzapine:  1. patients recovered the left dominant pattern of the electrical density in temporal regions, such as the Henschl gyrus; 2. improved performance on a test of verbal and learning memory; 3. The laterality index for patients after 6 months treatment with olanzapine was significantly larger than that at baseline indicating a shift of the high current source density areas in the temporal regions to the left side. |
| *Wichniak*  *2006* | - Highest percentage of patients with EEG abnormalities was found in the group co-treated with other AP, followed by the one treated with OLZ alone. The group of patients co-treated with BZD showed less EEG abnormalities and similar to untreated patients. Most frequent EEG abnormalities were slow waves. - Dose related increase in EEG abnormalities rate. - EEG abnormalities were not specifically localized to any brain area. |
| *Kikuchi*  *2007* | differences in microstates were observed comparing patients and control group, pre- and post-treatment in patient group.   - Patients had significantly more B and C and an overall increase of microstates occurrence, compared to controls; - Less percent total time spent in D in patients compared to controls; - Shorter duration of D and overall MS duration in patients compared to controls; - Patients had smaller GFS than controls in the theta band and a larger mean GFS in the gamma band. No differences in the other frequency bands - Negative correlation between change of total time in MS-B and risperidone equivalents at control EEG. - Treatment administration increases GFS of theta-band and decreases the one for alpha2-band. |
| *Khodayari-Rostamabad*  *2010* | / |
| *Ravan*  *2014* | / |
| *Mitra*  *2015* | - Significant difference between patients and controls at baseline on spectral power in gamma1 on the right frontal and left occipital cortex, gamma2 on the left frontal region, and gamma3 on the right and left parietal, left temporal and central regions. After corrections for multiple comparisons, significant differences persisted for left parietal and temporal gamma3 spectral power, and was higher in patients than in controls; - A significant and persistent reduction in spectral power values was observed on gamma1 and gamma2 bands in the left occipital regions. Right frontal gamma1 power showed significant reduction at week 4 compared to baseline, but showed also a significant increase at week 8 compared to week 4. Differences not resisting correction for multiple testing; - No relationship between AP type and dosage and PANSS score. |
| *Masychev*  *2020* | / |
| *Arikan*  *2021* | / |
| *Ciprian 2021* | / |
| *Dominicus 2023* | - After correction for multiple testing none of the comparisons between patients and controls showed significant differences - Random forest regression model application to the differentiation of patients and controls had an accuracy of 50.2% |
